# Riding the wetland wave: Can ducks locate macroinvertebrate resources across the breeding season?

**DOI:** 10.1002/ece3.11568

**Published:** 2024-06-25

**Authors:** Casey M. Setash, Adam C. Behney, James H. Gammonley, David N. Koons

**Affiliations:** ^1^ Colorado Parks and Wildlife Fort Collins Colorado USA; ^2^ Department of Fish, Wildlife, and Conservation Biology Colorado State University Fort Collins Colorado USA

**Keywords:** aquatic macroinvertebrates, drought, resource tracking, waterfowl, wetlands

## Abstract

Food availability varies considerably over space and time in wetland systems, and consumers must be able to track those changes during energetically‐demanding points in the life cycle like breeding. Resource tracking has been studied frequently among herbivores, but receives less attention among consumers of macroinvertebrates. We evaluated the change in resource availability across habitat types and time and the simultaneous density of waterfowl consumers throughout their breeding season in a high‐elevation, flood‐irrigated system. We also assessed whether the macroinvertebrate resource density better predicted waterfowl density across habitats, compared to consistency (i.e., temporal evenness) of the invertebrate resource or taxonomic richness. Resource density varied marginally across wetland types but was highest in basin wetlands (i.e., ponds) and peaked early in the breeding season, whereas it remained relatively low and stable in other wetland habitats. Breeding duck density was positively related to resource density, more so than temporal resource stability, for all species. Resource density was negatively related to duckling density, however. These results have the potential to not only elucidate mechanisms of habitat selection among breeding ducks in flood‐irrigated landscapes but also suggest there is not a consequential trade‐off to selecting wetland sites based on energy density versus temporal resource stability and that good‐quality wetland sites provide both.

## Graphical abstract

Nektonic macroinvertebrate density varies across wetland types and over time during the waterfowl breeding season, with basin wetlands harboring the highest density and the most stable resources. Waterfowl pairs are able to track these densities and are found in the greatest numbers in the wetlands with the highest food density during the breeding season.

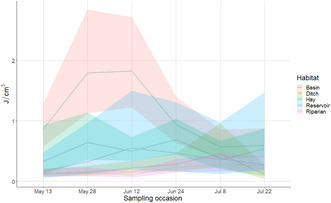

## 1 INTRODUCTION

The amount and availability of food resources are fundamental components of ecology and influence many decisions an animal makes. Consumers must find available resources during the correct timeframe and at sufficient quantities to survive and reproduce, with availability being a function of both resource phenology and the ability of the consumer to track that phenology (Abrahms et al., 2021). Consumers are impacted by multiple dimensions of resources, including the spatial variability across the landscape and limitations that inhibit the consumer from being ideal and free to track spatiotemporal variability in resource availability (e.g., defending a breeding territory, maintaining vigilance against predators, or rearing offspring; Baert et al., 2021; Behney et al., 2018; Fraser & Catlin, 2019; Fretwell & Lucas, 1970). Although animals may be forced to use suboptimal habitat (e.g., through despotic interactions), the degree to which animals use habitats relative to their availability should provide an indication of the processes shaping habitat quality (Clark & Shutler, 1999).

Some animals take advantage of pulses in resources throughout their annual cycles, often tracking these pulses through space and time (Armstrong et al., 2016). Some species even track resources during seasonal migrations (Evans & Bearhop, 2022; Tattoni et al., 2019). This concept has been popularized by the idea of a “green wave” among migratory herbivores, some of which closely track spring green‐up patterns of vegetation as they proceed toward the breeding grounds (Merkle et al., 2016; van der Graaf et al., 2006). The ability of some animals to track ephemeral resources has considerable fitness consequences, including both direct and indirect effects on survival and reproduction (Evans & Bearhop, 2022; Middleton et al., 2018). The synchrony of phenological events is especially important in systems where both the consumer demand and resource availability distributions are narrow, as is often the case in extremely seasonal habitats like those at high latitudes or elevation (Both et al., 2010; Lindén, 2018). Additionally, phenological mismatches are becoming increasingly common as climate change alters temporal resource distributions, migratory pathways and timing, and habitat conditions across the globe (Lawrence et al., 2022; Visser et al., 2012; Visser & Gienapp, 2019). Consumers that exploit transient pulses in resources during times of brief, but extreme nutrient demand is often at the highest risk of negative fitness consequences (Clark & Hobson, 2022; Kubelka et al., 2022; Simmonds et al., 2020). For example, insectivorous great tits (*Parus major*) rely on pulses of winter moth (*Operophtera brumata*) caterpillar abundance during the breeding season to successfully rear offspring and have been shown to exhibit thresholds in the plasticity of nest initiation with direct fitness consequences (Simmonds et al., 2020). Plasticity in foraging behavior and food specificity (i.e., a generalist foraging strategy) can thus be adaptive in terms of which resources can be exploited and which habitats can be used in the face of global change.

The extent to which consumers must expend energy tracking resources is influenced by the temporal variability in resource availability (i.e., the spread of the distribution of resource abundance over time and how much overlap there is with the distribution of consumer requirements). Habitats harboring consistent, abundant resources across the consumer's period of need likely have the highest probability of providing nutrients without requiring additional movements among habitats (Gurney et al., 2017; Pöysä et al., 2000). The exploitation of temporally stable resources may therefore provide an alternative mechanism explaining the patterns of observed consumer habitat use (i.e., bet‐hedging). When both resource pulses and temporally stable resources exist within a single system, preferences of consumers can be evaluated and inferences can be drawn about the predominant mechanisms driving foraging behaviors and habitat needs.

Breeding waterfowl, especially those toward the income end of the capital‐income spectrum of nutrient investment in breeding (Alisauskas & Ankney, 1992; Ankney & Alisauskas, 1991), commonly take advantage of pulses in aquatic invertebrate resources in order to produce offspring as those resources emerge and become available across variable habitats (Anteau, 2012; Drobney & Fredrickson, 1979; Gammonley & Laubhan, 2002; Stafford et al., 2016). Ducks have been shown to prospect for sites in advance of nesting that appear to provide high‐quality foraging habitat for themselves and their broods (Casazza et al., 2020; Eadie & Gauthier, 1985). Whether those sites remain high‐quality is a function of the diversity of the forage resource community, the degree to which that community pulses ephemerally, and a myriad of biotic and abiotic factors related to safety (Holopainen et al., 2024; Recer et al., 1987) and wetland plant configuration (Kaminski & Prince, 1981; Masto et al., 2022). Breeding waterfowl must be able to either select habitats where there is temporal stability in the resource or must be able to track resource pulses over space and time to ensure that their life‐history events overlap with the life stages at which resources are available (Deacy et al., 2018; Nummi et al., 2015). Dabbling duck females must acquire protein‐rich invertebrates to produce eggs and maintain their body condition throughout nest incubation (or at least minimize losses). Females must then allow their broods to maximize protein acquisition (predominately invertebrates) for offspring growth and survival alongside other macro and micronutrients (Gardarsson & Einarsson, 1994; Nummi et al., 2015; Paasivaara & Pöysä, 2008) as well as recover their own nutrient reserves lost during incubation (Cooper & Anderson, 1996; Krapu & Reinecke, 1992; Sedinger, 1992). Nest initiation, however, which is also a phenological trait selected for by environmental conditions, places a limit on the phenology of duckling hatch and habitat selection relative to their prey (de Szalay et al., 2003; Drever & Clark, 2007). The study of how ducks follow and exploit resource pulses within a season could elucidate potential mechanisms driving habitat selection, movement patterns, and fitness components (Drobney & Fredrickson, 1979; Gammonley & Laubhan, 2002; Murkin & Kadlec, 1986; Sjöberg et al., 2000).

The macroinvertebrate resources waterfowl exploit often remain in their various life stages ephemerally and have evolved developmental phenologies via top‐down (e.g., phenology of competitors and predators; Moore & Schindler, 2010) and bottom‐up selective forces (e.g., temporal shifts in water chemistry, temperature, and primary productivity; Whiles & Goldowitz, 2001). Developmental phenology of macroinvertebrates can vary among and within species depending on environmental conditions (McCauley et al. 2015). In terms of resources available to waterfowl consumers, differential invertebrate phenology may result in one or multiple resource pulses, or relatively consistent resource availability over time in a locally rich invertebrate community. Wetland habitats that are altered by humans, such as those in agricultural landscapes, may impact phenological drivers further, resulting in resource pulses that differ in magnitude and/or timing from naturally occurring wetlands. Ducks breeding in a matrix of natural and artificial wetlands may therefore face especially significant trade‐offs in habitat selection decisions, and their selection preferences or tracking abilities may imply advantages to restoring particular wetland types over others (Davis & Bidwell, 2008; Harrison et al., 2017; Wrubleski & Ross, 2011). Alternatively, a diverse matrix of both agricultural and naturally occurring wetlands in close vicinity to one another may present more opportunities to waterfowl by creating habitat for a diverse community of macroinvertebrates that emerge chronologically, thus providing consistent resources over time.

We evaluated factors affecting spatiotemporal variation in macroinvertebrate resource density and the extent to which breeding duck density was related to two metrics of resource availability across wetlands associated with flood‐irrigated agriculture. We predicted that ducks would exploit brief pulses in invertebrate resources, indicated by a positive correlation between waterfowl density and macroinvertebrate resource density across habitat types during the early nesting period (i.e., egg development period). Waterfowl are more mobile prior to nesting compared to when they are rearing broods, however, so we predicted brood densities would be more strongly related to temporal resource stability than to the phenology of absolute resource density.

## 2 METHODS

### 2.1 Study area

**FIGURE 1:**
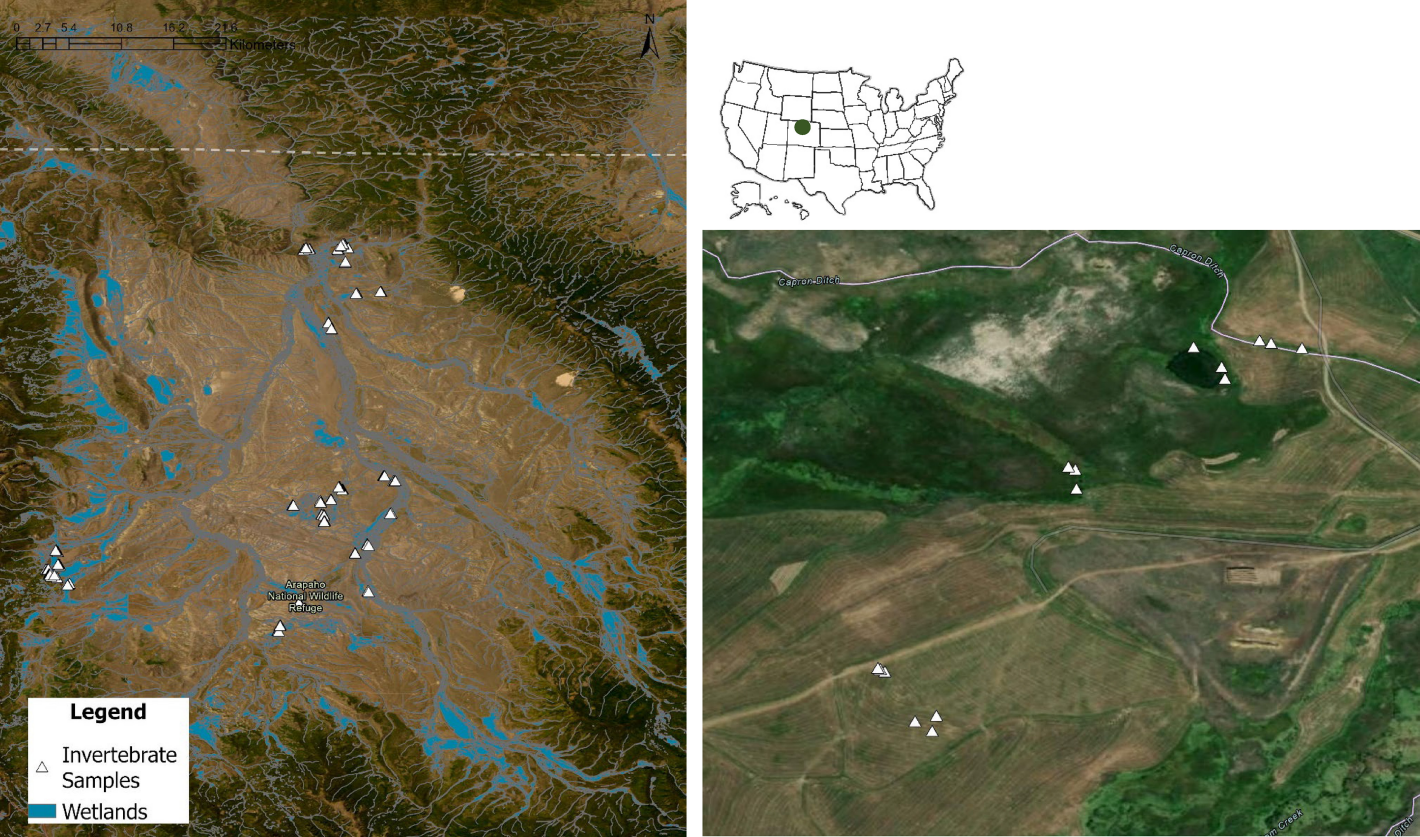
Map of wetlands and sampling locations across the study area in North Park, Colorado, USA from 2020 to 2021. We selected plots from four wetland types (basin wetland, riparian, irrigation ditch, and flooded hay meadow) on each of three properties to which we had access in addition to three public reservoirs in 2020 and 2021. The dashed line is the Wyoming‐Colorado border. We selected three random wetlands of each type on each of the three properties, and three random sampling points within each selected wetland with the exception of reservoirs. We randomly selected two 200‐m sections of shoreline in each reservoir and three random points within those “plots” to sample. The panel on the right shows an example of four different wetlands sampled on one of the properties. From bottom left to top right, the sampled points are in a flooded hay meadow, riparian wetland, basin wetland, and irrigation ditch. The three additional sampling points in the flooded hay meadow indicate that we moved those points in the second sampling year (2021) because the original locations were not flooded, which we accounted for in the analysis by treating them as different points nested in the same site.

Our study occurred during 2020–2021 throughout the North Platte Basin in Jackson County, Colorado, USA (North Park) along the North Platte River and its tributaries (Figure 1). North Park is a rural mosaic of natural and artificial wetlands resulting from flood‐irrigated hay agriculture. This high elevation (2600 m) basin comprising approximately 4300 km^2^ is dominated by salt desert shrubs and sagebrush steppe interspersed by lakes, ponds, irrigation ditches, irrigated hay fields, and the tributaries of the North Platte River. Land ownership is approximately 73% public, with the US Forest Service owning the largest parcels of public land (32%) that border the valley; however, 73% of wetland habitats in the study area are privately owned and associated with irrigated pastures and hay fields (Lemly & Gilligan, 2012). Most wetlands in the study area are located along linear, riparian corridors interspersed throughout the dominant upland shrub landscape (Figure 1). Arapaho National Wildlife Refuge (NWR), several State Wildlife Areas, Bureau of Land Management properties, and privately irrigated fields encompass many of the wetlands available to breeding and migrating waterfowl in the region and especially in the state of Colorado. Wetland habitats encompassed by this study included reservoirs, basin wetlands (i.e., ponds), irrigation ditches, flooded hay meadows, and riparian areas (Lemly & Gilligan, 2012). We defined reservoirs as permanent, artificial wetlands typically 1–8 m deep with a total flooded area >30 ha and <10% emergent vegetation. Basin wetlands were smaller, shallower, semi‐permanent wetlands with 15%–74% emergent vegetation. Irrigation ditches were artificially‐ created canals ranging from 1 to 3 m wide and 0.14–3.4 ha in total flooded area, lined with hard‐packed substrate, and typically bordered by graminoid vegetation rather than willows, whereas riparian sites were braided stream channels bordered by dense willows, lined with rocks or looser substrate, and ranging from 3 to 6 m wide. Flooded hay meadows were flat graminoid meadows 0.72–16 ha in flooded area with 75%–100% emergent vegetation. Hay meadows primarily consist of Timothy grass (*Phleum pretense*) interspersed with sedges (Cyperaceae) and rushes (Juncaceae).

This and other intermountain basins across western North America provide regionally‐important wetland habitat to breeding and migrating ducks. For example, of the 693 female locally‐hatched (i.e., flightless when captured) or hatch‐year (i.e., flighted when captured) mallards banded in North Park from 2018 to 2023, 9% have been recovered by hunters and 73% of those recoveries were in Colorado, suggesting the area is an important contributor to the regional fall flight of ducks (Gammonley, unpublished data).

### 2.2 Macroinvertebrate data collection

We collected nektonic invertebrate samples using 2‐L activity traps in 2020 and 2021 (Murkin et al., 1983). We placed traps at randomly selected points within 40 wetland sites that encompassed the five different wetland habitats. The sites we sampled in four of the wetland habitats (basin wetlands, riparian wetlands, irrigation ditches, and flooded hay meadows) spanned three individual properties, including two private ranches and Arapaho NWR (Figure 1). We also sampled three public reservoirs across the study area. We selected three random wetlands of each type on each of the three properties, and three random sampling points within each selected wetland with the exception of reservoirs. We randomly selected two 200‐m length plots of shoreline in each reservoir and three random points within each plot to sample (Behney, 2020a, 2020b; Cooper & Anderson, 1996; de Szalay et al., 2003). In total, we deployed 126 traps during each sampling occasion (3 properties × 4 wetland habitats × 3 sampled wetland sites of each variety × 3 sampled points in each wetland) = 108 samples, + (3 reservoirs × 2 plots in each reservoir × 3 points in each plot) = 18 samples. Traps remained in the wetlands for 48 h every 14 days, resulting in six sampling occasions each year over the course of the breeding season (13 May through 22 July). No traps were placed if the wetland was dry on a given sampling occasion, and that status was noted and aquatic invertebrate resource density was treated as a zero for that occasion. In the event that a wetland was not flooded or less flooded during the second year of sampling (2021), we randomly selected new points within the new boundary of the same wetland and treated those sampling points as unique from the original locations, but nested within the same site. Occasionally, traps became dislodged and either went missing or floated to the surface, in which case we replaced traps and allowed them to remain in the wetland for the subsequent 48 hours.

Activity traps had a 15 cm opening at the widest part of the funnel and a 2 cm opening at the narrowest part of the funnel. We placed them so that the top of the widest part of the funnel was approximately 1 cm above the surface of the water to capture invertebrates in the part of the water column in which dabbling ducks most often forage (Behney, 2020a, 2020b; Guillemain et al., 2000). Upon collection, traps were emptied into a mesh sieve‐bottom bucket. All individual invertebrates from the sample were placed into plastic storage cups and stored in 70% ethanol until processing. We emptied each sample into a 0.355 mm (number 45) gauge mesh sieve in a wet lab and moved all individuals to a Petri dish for identification and counting (Behney, 2020a, 2020b). We placed samples under a dissecting microscope (AmScope SM‐1BSY‐64S Stereo Zoom Microscope) and identified individuals to taxonomic Family when possible. Any sample containing more than 1000 individuals of a given Family was subsampled by 16.6% using a 6 × 6 square gridded Petri dish (Behney, 2020a, 2020b; Williams et al., 2014). We counted individuals in a random subset of six of the 36 cells and multiplied by six to estimate the total number of individuals of that Family in the sample.

### 2.3 Waterfowl data collection

We conducted breeding pair counts of ducks on the same wetland sites being sampled for macroinvertebrates (*n* = 40) using a dependent double‐observer methodology during the breeding seasons of 2020 and 2021 (Nichols et al., 2000). Pair count survey timing coincided with the first three macroinvertebrate sampling occasions. A primary observer counted every individual dabbling and diving duck observed and reported the number to a secondary observer, who recorded data while also recording any observations missed by the primary observer (Roy et al., 2021). We restricted the dataset to the four most common species of breeding ducks in our study system, which included cinnamon teal (*Spatula cyanoptera*), gadwall (*Mareca strepera*), mallard (*Anas platyrhynchos*), and lesser scaup (*Aythya affinis*). Although we used the standard pair count practice of separating lone drakes from paired ducks in each count to identify breeding phenology and thus the timing of our first invertebrate sampling occasion, we used the total count of breeding ducks for the purposes of evaluating the relationship between duck density and invertebrate availability. In addition, few individuals were missed by the primary observer (i.e., detection probability was high), so we pooled observed drakes and hens of a given species to give us a site‐ and occasion‐specific count.

Brood surveys also occurred on the sites sampled for macroinvertebrates, but followed an independent double‐observer methodology during 2020–2021 (Nichols et al., 2000; Vrtiska & Powell, 2011). Both observers counted the number of ducklings they observed and subsequently compared observations to determine whether they had been observing the same brood and compare the number counted. The smaller number of ducklings commonly observed at one time allowed for accurate count comparisons between observers (Pagano & Arnold, 2009). The timing of brood counts coincided with the latter three macroinvertebrate sampling occasions. Observers counted all ducklings within a given brood and identified their age class according to Gollop and Marshall (1954). Observers spent a minimum of 10 min at each wetland site and conducted surveys using window‐ or tripod‐mounted spotting scopes and binoculars to allow time for hidden broods to become visible (Pagano & Arnold, 2009; Walker et al., 2013). We restricted the dataset to ducklings of the same four duck species listed above and pooled the total number of ducklings for a given species, site, and occasion.

### 2.4 Macroinvertebrate analyses

We used a subset of the dataset to evaluate only the densities of macroinvertebrates commonly found in esophageal contents of ducks in past diet studies (see citations in Table 1). We searched the literature to find evidence indicating whether each observed Family could be considered a common waterfowl food item and the average energy density associated with a given individual of each selected family (kcal/g; Nudds & Bowlby, 1984). Breeding ducks rely on invertebrates primarily as a source of protein and various micronutrients (e.g., calcium; Swanson, 1984) that are needed for egg formation, duckling growth, and feather replacement during molt, but detailed information on protein and micronutrient content is not available for all aquatic invertebrate taxa. Therefore, we used more widely available average energy values for various invertebrate taxa as a surrogate for wetland forage value to breeding waterfowl. Using an average energy density rather than weighing the dry mass of each taxon in each sample saved considerable sample processing time and allowed us to process thousands of samples over the course of two years. The selected subset of taxa comprised 69.4% of all individual invertebrates sampled, suggesting it represented the majority of invertebrates present at our sites (see Appendix S1). We multiplied the energy density of each Family by the mean mass of an individual associated with that family in grams, resulting in the average kcal/individual (Table 1). These energy densities multiplied by the number of individuals of each Family observed in a given sample resulted in an estimate of kcal/sample, which we converted to kcal/cm^3^ of water sampled and then joules (J)/cm^3^. We also computed the number of distinct taxa classified as common duck foods (hereafter: taxonomic richness) identified in each sample. A challenge with duck food studies is the lack of reliable information on specific invertebrate taxa preferred by ducks (Klimas et al., 2022). Although ducks likely prefer some taxa within our selected subset, without preference information, we believe combining taxa representing known food items into an overall energy density metric was the best way to capture the value of a wetland to ducks in terms of the invertebrate resource. Additionally, the taxonomic richness covariate we included nicely accounts for some of that uncertainty in food preference and community composition.

**TABLE 1:** Macroinvertebrate families observed in wetland samples taken from 2020 to 2021 in North Park, Colorado that were considered important food items for breeding waterfowl (Bartonek & Murdy, 1970; de Szalay and Resh, 1997; de Szalay et al., 2003; Eldridge, 1990; McCutchen & Ydenberg, 2005; Myers, 1982).

Order	Family	Common name	Mean mass (g)	Mass citation	kcal/g Nudds and Bowlby (1984)	Mean/median/SD invertebrate density (individuals/L)
Amphipoda	Gammaridae	Scuds	0.0105	Driver et al. (1974)	2.32	23.4/2.5/102.6
Anostraca	Chirocephalidae	Fairy Shrimp	0.00037	Hildrew (1985)	5	5.40/3.75/5.06
Calanoida	Diaptomidae	Copepods	3.30E‐12	Stead et al. (2003)	4.96	11.5/3/23.5
Cladocera	Daphniidae	Water Fleas	0.00493	Dumont et al. (1975)	4.8	180/5/1523
Coleoptera	Dytiscidae	Predaceous Diving Beetles	0.013167	Driver et al. (1974)	5.3	5.78/2.5/9.93
Coleoptera	Hydrophilidae	Water Scavenger Beetles	0.013167	Driver et al. (1974)	5.6	1.86/1/2.45
Diptera	Ceratopogonidae	Biting Midges	0.0045	Driver et al. (1974)	5.2	1.58/0.5/1.94
Diptera	Chironomidae	Non‐Biting Midges	0.0045	Driver et al. (1974)	5.5	2.80/1/6.59
Diptera	Culicidae	Mosquitoes	0.0058	Driver et al. (1974)	5.2	29.1/1/185
Diptera	Simuliidae	Black Flies	0.0045	Driver et al. (1974)	5.2	20.2/1/92.0
Gastropoda	Ancylidae	Limpets	0.0367	Driver et al. (1974)	1	0.5/0.5/NA
Gastropoda	Lymnaeidae	Pond Snails	0.0367	Driver et al. (1974)	1	1.61/1/2.29
Gastropoda	Physidae	Pouch Snails	0.0367	Driver et al. (1974)	1	1.09/0.5/1.02
Gastropoda	Planorbidae	Orb Snails	0.0367	Driver et al. (1974)	1	1.27/0.5/1.31
Hemiptera	Corixidae	Water Boatmen	0.0033	Driver et al. (1974)	5.5	7.96/1.50/26.9
Ostracoda	Ostracoda	Seed Shrimp	0.000282	Stead et al. (2003)	5	199/4.50/1058
Plecoptera	Chloroperlidae	Green Stoneflies	0.034833	Allan (1982)	5.5	0.60/0.50/0.22
Plecoptera	Perlodidae	Stripetail Stoneflies	0.034833	Allan (1982)	5.5	0.50/0.50/0
Plecoptera	Plecoptera	Unknown Stoneflies	0.034833	Allan (1982)	5.5	0.90/0.50/1.41
Trichoptera	Brachycentridae	Humpless Casemaker Caddisflies	0.011618	Driver et al. (1974)	5.4	1.27/0.50/1.79
Trichoptera	Lepidostomatidae	Scaly‐Mouth Caddisflies	0.011618	Driver et al. (1974)	5.4	0.70/0.50/0.27
Trichoptera	Leptoceridae	Long‐Horned Caddisflies	0.011618	Driver et al. (1974)	5.4	0.83/0.75/0.41
Trichoptera	Limnephilidae	Northern Caddisflies	0.011618	Driver et al. (1974)	5.4	1.09/0.50/1.18
Trichoptera	Odontoceridae	Mortarjoint Casemaker Caddisflies	0.011618	Driver et al. (1974)	5.4	0.50/0.50/0
Trichoptera	Phryganeidae	Giant Casemaker Caddisflies	0.011618	Driver et al. (1974)	5.4	0.91/0.50/0.63
Trichoptera	Polycentropodidae	Tube‐Making Caddisflies	0.011618	Driver et al. (1974)	5.4	1.60/1/1.19
Trichoptera	Trichoptera	Unknown Caddisflies	0.011618	Driver et al. (1974)	5.4	1.27/0.50/1.79
Trombidiformes	Hydrachnidia	Water Mites	0.0148	Driver et al. (1974)	5.6	4.19/1.50/12.3

Temporal resource stability has been evaluated using a myriad of methodologies. We chose to use a common approach in the community ecology literature, the Species Rank Abundance Curve (MacArthur, 1957; Whittaker, 1965), and applied it to the energy density distribution across sampling occasions to evaluate the “evenness” of resources over time. This method accounted for the non‐normal distribution of energy density over time and encompasses the relative amount of nutrients available in addition to whether and how quickly that changes over time. For a given wetland site, sampling occasion, and year, we took the median of the energy density from the sample replicates at that site. For each site‐year combination, we ranked each sampling occasion by the average energy density (J/cm^3^), creating an energy rank abundance curve for each site, each year. From these curves, we calculated a metric of evenness, *E*_Q_, which is a measure of the slope of the curves and thus how quickly energy changes (Avolio et al., 2019). A higher *E*_Q_ indicates a more even/stable resource for the consumer within a given site and year, and a higher y‐intercept indicates higher overall resource density relative to other sites. We used each site‐ and year‐specific *E*_Q_ value as a predictor of duck density in the next stage of the analysis. For visualization purposes, we also condensed energy density across sampled sites to examine habitat‐ and year‐specific variation in resource stability. Again, we used the median energy density associated with a given habitat type and sampling occasion to create a habitat‐specific rank abundance curve and compute the *E*_Q_ metric.

The first stage of the analysis was to model energy density over time for each habitat type. Energy data were strictly positive with true zeros occurring in dry wetlands. We therefore fit a lognormal hurdle model to energy (i.e., nutrient) density over the sampling period to evaluate drivers of temporal shifts in resource density (Feng, 2021; Morrow et al., 2015). Fixed effects included categorical terms for wetland habitat, sampling occasion, and an interaction term of habitat × sampling occasion (Gammonley & Laubhan, 2002; Stafford et al., 2016), wetland size (ha; continuous), a term for taxonomic richness of invertebrates in the sample (continuous), and a quadratic effect of taxonomic richness (Gough et al., 1994). The quadratic term was included to evaluate whether energy density was highest at intermediate richness levels (Gross et al., 2014). We included a habitat fixed effect on the Bernoulli process determining whether a given site had a nonzero energy density in addition to a binary indicator of whether the site was dry when we sampled it. We provided vague priors for all parameters and standardized the continuous wetland size covariate (Hobbs & Hooten, 2015). We predicted energy density for each habitat‐occasion combination to visualize the phenological shifts of energy density in the system and make inference on the drivers of those shifts. We also fit a second model including site, sampling occasion, and year as fixed effects, and an interaction term between the three. This allowed us to predict energy density at each site‐occasion‐year combination to use as a predictor of waterfowl density in subsequent models.

### 2.5 Waterfowl analyses

We processed data to obtain a count of species‐specific duck abundance at each site‐occasion‐year combination after migration had concluded for our four focal species, cinnamon teal, gadwall, mallard, and lesser scaup. We then standardized abundance by wetland size to obtain a density estimate (ducks/ha), which we rounded up to the next highest integer in order to fit a zero‐inflated Poisson model (Feng, 2021). Using a logit link function, we fit a model with a categorical habitat predictor on the zero inflation parameter (Ψ), which indicates the probability that a sampled site is perceived as unavailable habitat for ducks or ducklings (Zipkin et al., 2014). We fit four models corresponding to specific mechanistic hypotheses on the intensity parameter of the Poisson component of the ZIP (*λ*) using a log link: an intercept‐only null model, a model including a categorical sampling occasion predictor (*β*_week_) to evaluate whether duck density changed over time in a fashion unrelated to macroinvertebrates, a model with an invertebrate energy density covariate (*β*_energy_), and a model with the temporal resource evenness (*E*_Q_) predictor, as described above. To fully propagate uncertainty in predicted invertebrate energy density to this stage of the analysis, we drew each site‐, occasion‐, and year‐specific value of energy density from a lognormal distribution where the mean and standard deviation were assigned from the model‐predicted values resulting from the first stage of the analysis (Behney, 2020a, 2020b; Gilbert et al., 2023).

We repeated these analyses using species‐specific duckling density (ducklings/ha) as a response variable to evaluate resource tracking across the full breeding cycle (where the latter three invertebrate sampling occasions were used to inform the energy density explanatory variable). There were few mallard and cinnamon teal duckling detections in sites sampled for macroinvertebrates, resulting in convergence issues for single‐species models. We therefore pooled these two species into an “early nester” category, while gadwall and lesser scaup duckling densities were evaluated separately. Mallards and cinnamon teal, while exhibiting different life‐history strategies, have shown preferences for similar habitats, if not invertebrate food sources (Hohman & Ankney, 1994; Mackell et al., 2021), initiated nests at similar times (30 May for mallards [SD = 17 days], 2 June for cinnamon teal [SD = 13 days]), and are both on the fast end of the slow‐fast life‐history spectrum (Koons et al., 2014).

We conducted all analyses in a Bayesian framework using the jagsUI package in Program R (Kellner, 2024). We implemented the lognormal hurdle model using the “zeroes trick”, which allows a user to specify custom sampling distributions (Hilbe et al., 2017; Spiegelhalter et al., 2003). We ran three chains for 40,000 iterations, a burn‐in period of 5000 iterations, and kept every third iteration to thin the chains. We specified vague priors for all parameters on the appropriate link scale (Northrup & Gerber, 2018). We checked for convergence visually using trace plots and evaluated Gelman‐Rubin statistics, ensuring all were ≤1.1 (Gelman & Rubin, 1992; Hobbs & Hooten, 2015). We provide posterior means and standard deviations, as well as the proportion of the posterior that was on the same side of 0 as the mean for each parameter, denoted as *f* (Buderman et al., 2023). We present 95% highest posterior density intervals (HPDI) in all applicable figures.

## 3 RESULTS

We collected 778 site‐ and occasion‐specific invertebrate samples across 40 wetland sites in 2020 and 668 samples across the same 40 sites in 2021. Of the total samples, 171 and 147 were from wetlands that were dry on a given sampling occasion in 2020 or 2021, respectively. We identified a total of 114 unique invertebrate taxa at the lowest level of identification possible (mostly Families, but some Superfamilies, Orders, and Classes; Table S1) and used a subset of 28 Families that are commonly known to be eaten by waterfowl in our analyses (Table 1). Taxonomic richness ranged from 1 to 12 (mean = 3.62, SD = 2.55) within a given sample and the taxa comprising the greatest proportion of the total number of invertebrate individuals and the total energy density provided by invertebrates were most commonly Daphniidae and Ostracoda (Figures S1 and S2).

We counted the largest number of ducks in reservoirs and basin wetlands, totaling 676 and 786 in basins and reservoirs in 2020, respectively, and 880 and 2304 in basins and reservoirs in 2021. We observed 48 and 62 ducks in riparian habitats in 2020, and 2021, respectively, 17 and 13 in hay meadows, and 19 and 13 in irrigation ditches. Mallards comprised the largest proportion of pair counts, ranging from 0.25 of reservoir counts in 2020 to the only species (proportion = 1.00) observed in ditches and hay meadows in 2021 (Table S2). Gadwall broods made up the highest proportion of ducklings observed in most habitats; however, ranging from 0.17 of broods in basins in 2021 to 0.71 of broods in basins in 2020 (Table S3). No ducklings of any species were observed in irrigation ditches or hay meadows, and the largest number of observed ducklings was in reservoirs and basin wetlands in both years (340 in reservoirs in 2020 and 76 in 2021, 102 in basins in 2020 and 89 in 2021). In riparian habitats, 16 and 9 ducklings were observed in 2020 and 2021, respectively.

### 3.1 Drivers of resource density and stability

**FIGURE 2:**
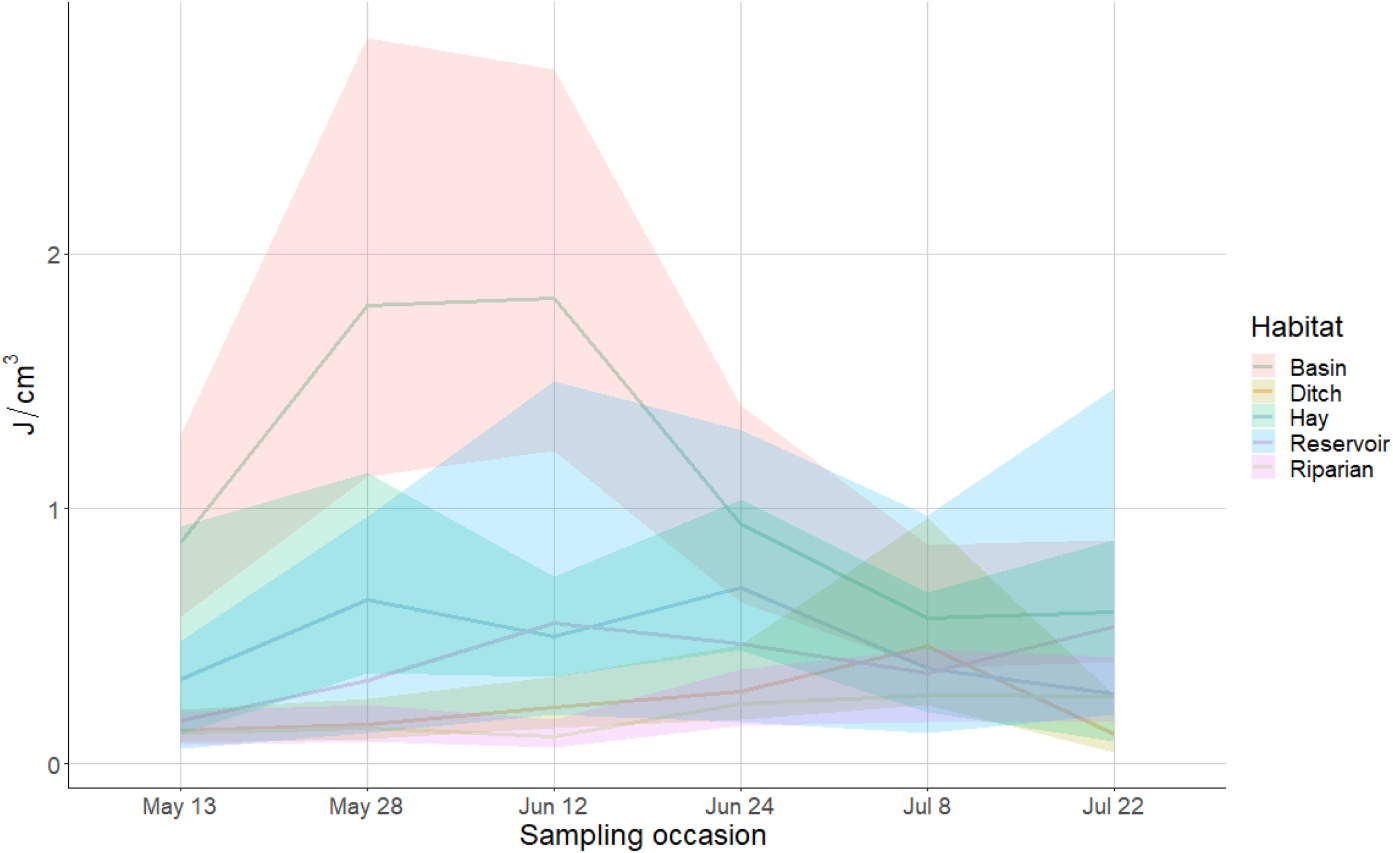
Model‐predicted estimates of invertebrate energy density (J/cm^3^) across wetland habitats throughout the breeding season (May–July) in North Park, CO, 2020–2021.

**FIGURE 3:**
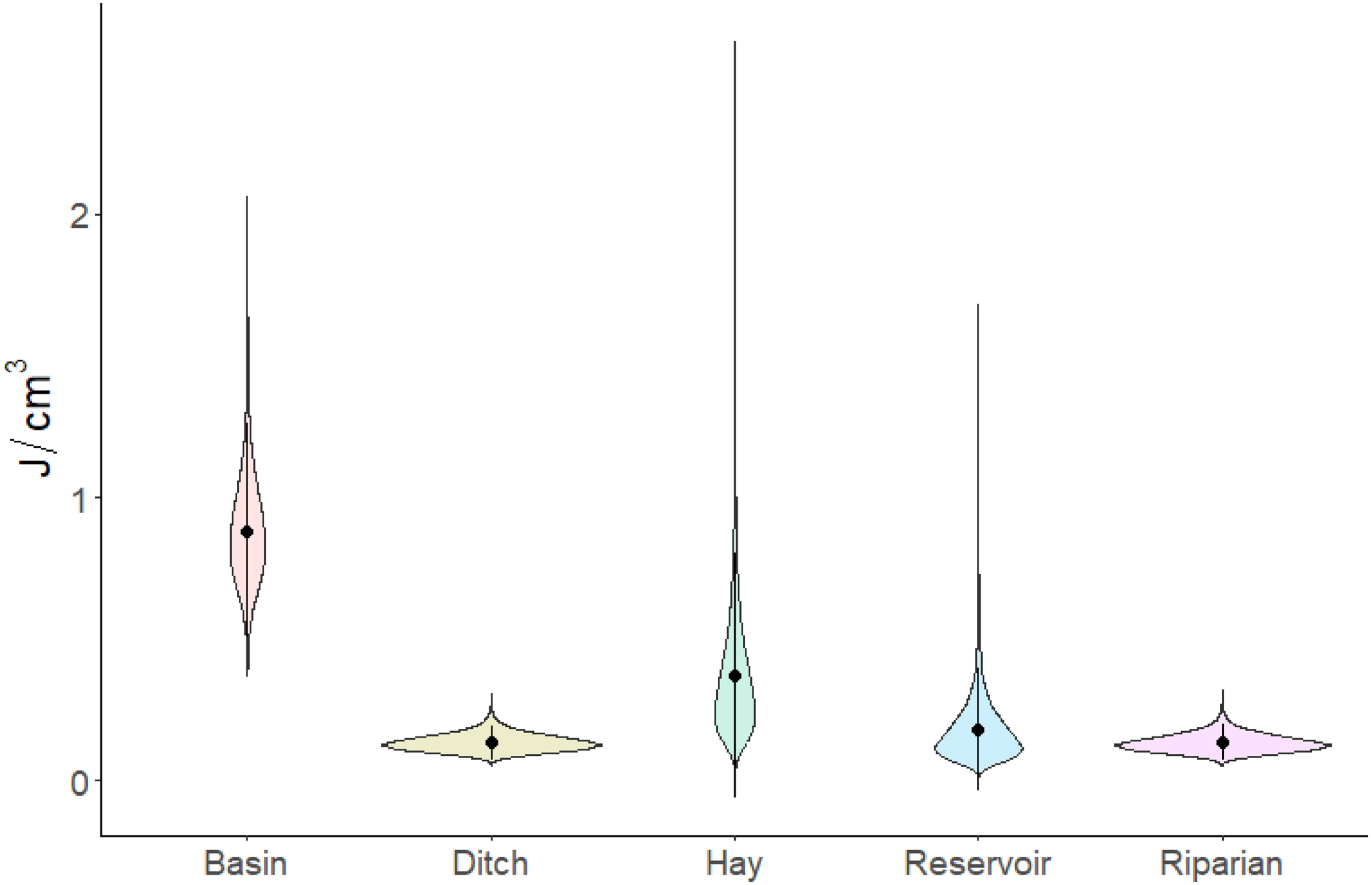
Time‐averaged invertebrate energy density (J/cm^3^) available in each sampled wetland habitat in North Park, CO from 2020 to 2021. Points represent posterior means of the habitat‐specific energy density, bars represent 95% credible intervals, and violin shapes represent the posterior distribution to provide a reference of the amount of sampling variability in each habitat type.

**FIGURE 4:**
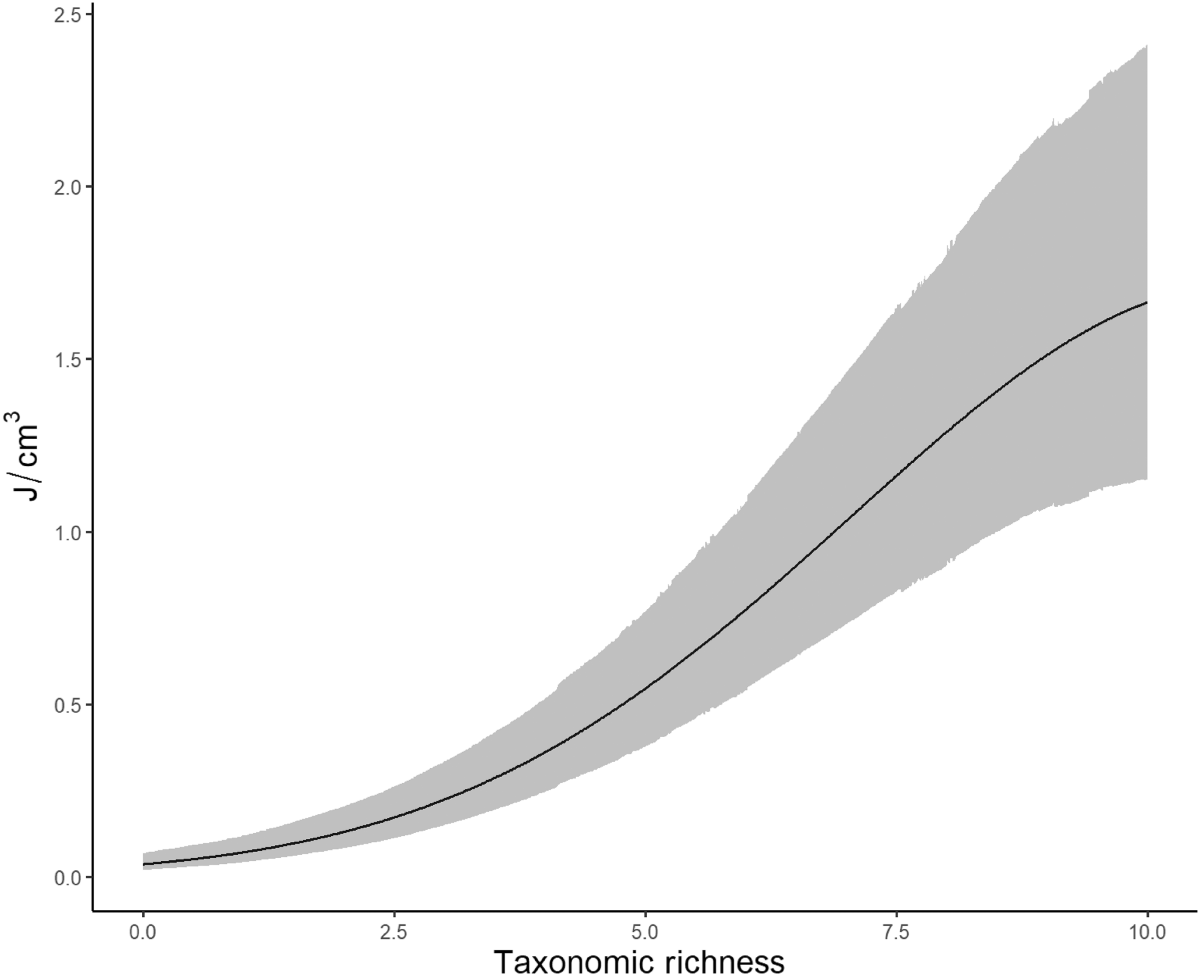
Model‐predicted energy density (J/cm^3^) as a function of observed taxonomic richness (number of taxa) of macroinvertebrates sampled in North Park, CO, USA.

Macroinvertebrate energy density varied by habitat and over the course of the breeding season (Figures 2 and 3, Table S4). Basin wetlands exhibited the only pulse in energy resources of any wetland type, with energy in basins peaking early in the season and subsiding later, at which time they exhibited energy density comparable to other habitat types (Figure 2). Invertebrate energy density in other wetland types did not vary considerably over time, instead remaining consistently low. Flood‐irrigated hay meadows pulsed higher in energy density than reservoirs on two sampling occasions, but otherwise contained relatively low energy density (Figure 2). Higher energy density was associated with larger wetlands (*β* = 0.58, SD = 0.25), and there was support for a positive relationship between energy density and taxonomic richness (*β*_rich_ = 1.21, SD = 0.06; *β*_rich2_ = −0.29, SD = 0.04; Figure 4).

**FIGURE 5:**
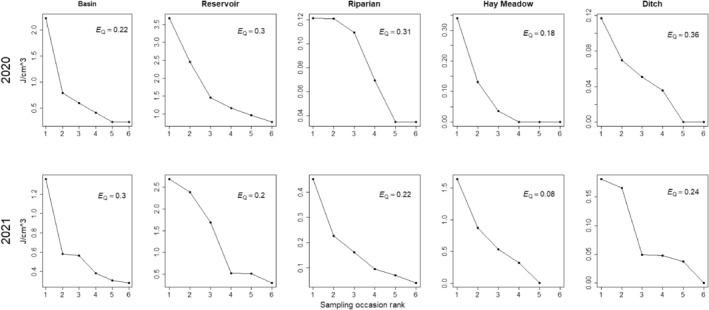
Temporal rank abundance curves created by ranking the energy density (J/cm^3^) of a given wetland type across six sampling occasions in 2020 and 2021, North Park, CO, USA. *E*_Q_ provides a metric of the evenness of a resource as measured by the slope of a rank abundance curve (high *E*_Q_ indicates higher evenness of the resource over time).

The evenness metric we computed, *E*_Q_, varied from 0.01 to 0.50 across sites (mean = 0.19, SD = 0.09). Site‐averaged, habitat‐specific measures of *E*_Q_ varied from 0.08 in hay meadows (2021) to 0.36 in irrigation ditches (2020; Figure 5). Habitat types associated with relatively low energy density (e.g., irrigation ditches and riparian wetlands; Figure 2) typically had high *E*_Q_ values, indicating that there were few or no pulses in invertebrate energy availability.

### 3.2 Waterfowl density

**FIGURE 6:**
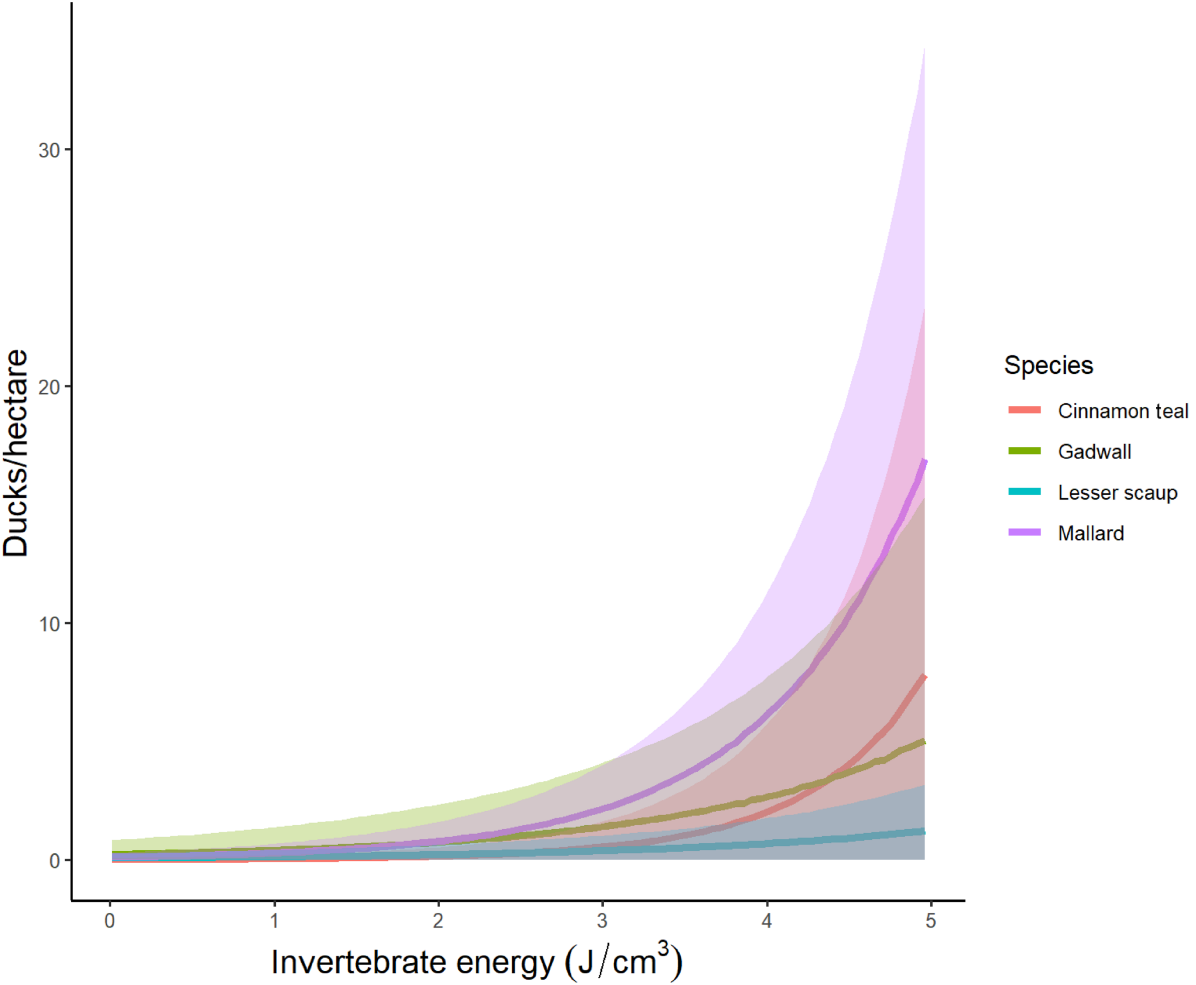
Relationship between breeding duck density and invertebrate energy density (J/cm^3^) for each duck species across wetlands in North Park, CO, USA from 2020 to 2021.

The average duck density per survey across habitats and years varied from 0.18 ducks/ha in hay meadows (2021) to 9.49 ducks/ha in basin wetlands (2021). Duckling density also varied by habitat type, ranging from 0 in both hay meadows and ditches (2020 and 2021) to 0.90 ducklings/ha in riparian wetlands (2020). The model including energy density (J/cm^3^) as a predictor of breeding duck density (ducks/ha) performed better than the null model, the model including sampling occasion, the model of temporal resource stability (*E*_Q_), and the model including taxonomic richness for all duck species (Table 2). Duck density exhibited a positive relationship with energy density across focal duck species. Cinnamon teal responded most strongly to energy density (*β*_energyCITE_ = 1.98, *σ*_energyCITE_ = 0.38, *f*_energyCITE_ = 0.94), followed by mallard (*β*_energyMALL_ = 1.50, *σ*_energyMALL_ = 0.21, *f*_energyMALL_ = 0.98), gadwall (*β*_energyGADW_ = 0.94, *σ*_energyGADW_ = 0.20, *f*_energyGADW_ = 1), and lesser scaup (*β*_energyLESC_ = 0.86, *σ*_energyLESC_ = 0.19, *f*_energyLESC_ = 0.98; see Figure 6). These coefficients mean that, among wetlands which individuals of a given species perceive as acceptable for use, a one J/cm^3^ increase in energy density led to an increase in the average number of ducks/ha by a factor of exp(1.98) = 7.45 for cinnamon teal, 4.45 for mallards, 2.57 for gadwall, and 2.35 for lesser scaup. However, these coefficients were moderated by the probabilities that a given wetland habitat held zero ducks, which were highest for gadwall and lesser scaup, but higher for cinnamon teal than mallards across wetland habitat types (Table 3).

**TABLE 2:** WAIC values associated with models of breeding duck density or duckling density in North Park, Colorado, USA from 2020 to 2021.

Response variable	Model/hypothesis	WAIC
Mallard density	Energy density	394
	Sampling occasion	812
	Null	823
	Taxonomic richness	829
	Temporal evenness	830
Cinnamon teal density	Energy density	184
	Taxonomic richness	300
	Temporal evenness	362
	Null	363
	Sampling occasion	370
Gadwall density	Energy density	204
	Taxonomic richness	324
	Temporal evenness	346
	Sampling occasion	360
	Null	363
Lesser scaup density	Energy density	116
	Null	153
	Temporal evenness	154
	Taxonomic richness	156
	Sampling occasion	158
Mallard/cinnamon teal duckling density	Energy density	119
	Null	144
	Sampling occasion	146
	Taxonomic richness	149
	Temporal evenness	166
Gadwall duckling density	Taxonomic richness	95
	Energy density	100
	Null	101
	Temporal evenness	105
	Sampling occasion	106
Lesser scaup duckling density	Energy density	59
	Temporal evenness	60
	Null	60
	Sampling occasion	64
	Taxonomic richness	73

*Note*: Models are sorted by WAIC value from lowest (best model) to highest for each response variable. We modeled duck or duckling density for each species using a zero‐inflated Poisson model where the intensity parameter (*λ*) was a function of macroinvertebrate resource density (J/cm^3^; energy density) when the counts occurred (sampling occasion), an intercept‐only null model, taxonomic richness of invertebrate food resources (taxonomic richness), and a metric of the resource stability stemming from a rank abundance curve (temporal evenness).

**TABLE 3:** The probability (SD) that a given wetland habitat held zero ducks for each species grouping in wetlands sampled in North Park, CO, USA from 2020 to 2021.

Species	Basin	Irrigation ditch	Hay meadow	Reservoir	Riparian
Cinnamon teal adults	0.43 (0.11)	0.73 (0.15)	0.68 (0.16)	0.09 (0.09)	0.67 (0.17)
Mallard adults	0.26 (0.08)	0.72 (0.09)	0.44 (0.15)	0.06 (0.06)	0.17 (0.12)
Gadwall adults	0.58 (0.09)	0.93 (0.04)	0.88 (0.08)	0.08 (0.07)	0.83 (0.07)
Lesser scaup adults	0.47 (0.11)	0.94 (0.06)	0.88 (0.11)	0.07 (0.07)	0.93 (0.07)
Cinnamon teal/mallard ducklings	0.84 (0.07)	0.96 (0.06)	0.90 (0.16)	0.36 (0.14)	0.93 (0.04)
Gadwall ducklings	0.76 (0.07)	0.94 (0.07)	0.87 (0.16)	0.45 (0.13)	0.94 (0.04)
Lesser scaup ducklings	0.78 (0.11)	0.92 (0.12)	0.84 (0.21)	0.57 (0.19)	0.97 (0.04)

*Note*: Probabilities were estimated using the top model for each species grouping from the binomial component of a zero‐inflated Poisson model.

**FIGURE 7:**
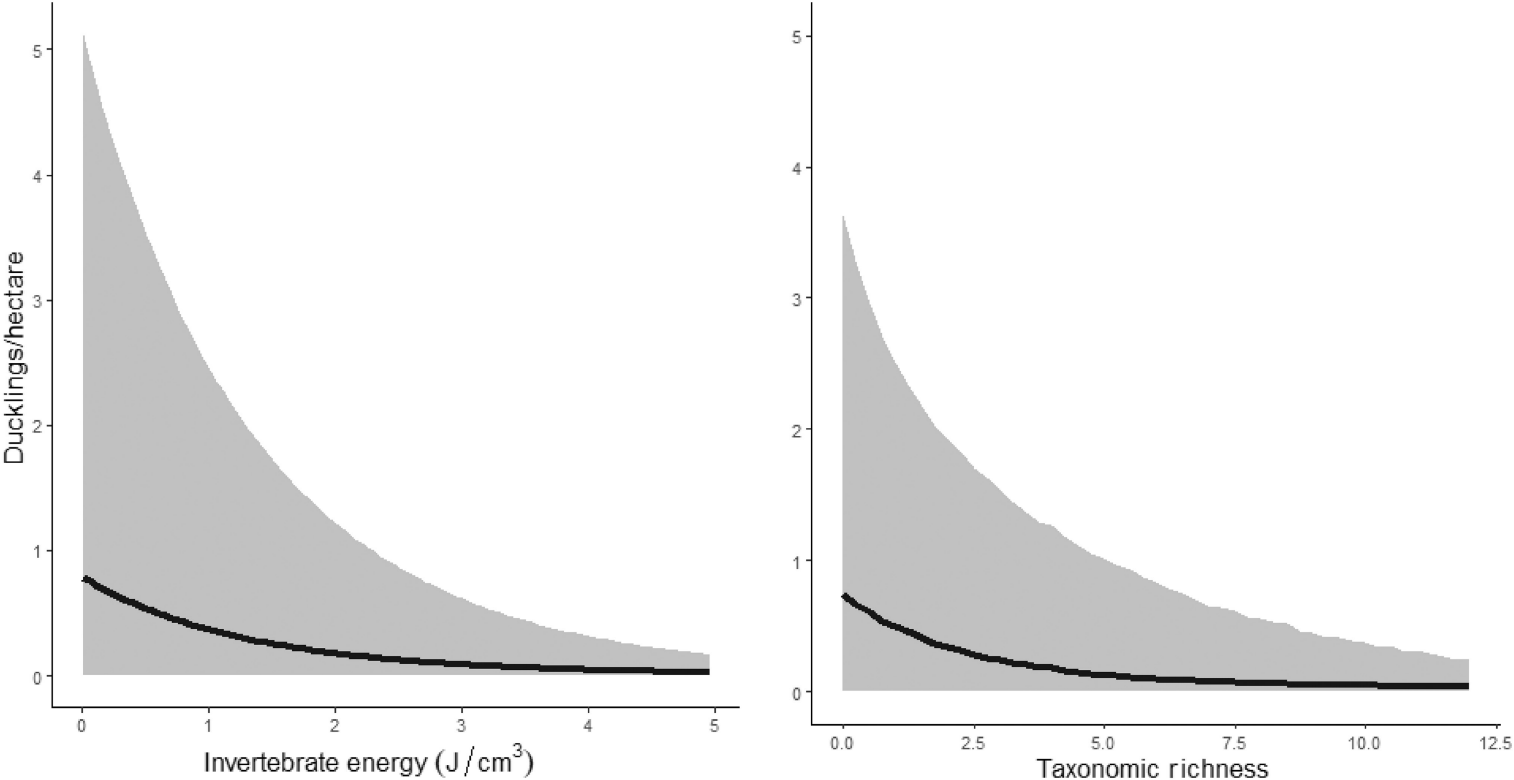
Relationship between duckling density and the invertebrate predictor in the top model as measured by WAIC across wetlands in North Park, CO, USA from 2020 to 2021. The left panel is the relationship between pooled mallard and cinnamon teal duckling density with energy density (J/cm^3^), and the right panel is the relationship between gadwall duckling density and taxonomic richness.

Duckling density exhibited different patterns with invertebrate resources than breeding duck density. The model including energy density performed marginally better than all other models for the “early nester” group (i.e., mallards and cinnamon teal) and lesser scaup (Table 2). Both groups exhibited a negative relationship with energy density (*β*_energy,MALL/CITEducklings_ = −1.10, *σ*_energy,MALL/CITEducklings_ = 0.27, *f*_CITE/MALLducklings_ = 1; *β*_energy,LESCducklings_ = −0.52, *σ*_energy,LESCducklings_ = 1.08, *f*_LESCducklings_ = 0.84; Figure 7) although the relationship was less precise for lesser scaup. The taxonomic richness model was the best fit for gadwall duckling density, and the relationship was also negative (*β*_energy,GADWducklings_ = −0.40, *σ*_energy,GADWducklings_ = 0.16, *f*_GADWducklings_ = 0.99; Figure 7). The probability that a wetland held zero ducklings was lowest for reservoirs across species and typically higher for the early nester group (Table 3). Despite better model performance compared to a null model using WAIC, effect sizes of invertebrate predictors on duckling density were relatively small, and model‐predicted mean duckling density varied from 0.02 to 0.79 ducklings/ha across the range of sampled energy density or taxonomic richness values.

## 4 DISCUSSION

The mechanisms driving breeding habitat selection and resource tracking are complex and vary across species and ecosystems. In an ecosystem characterized by highly variable water availability and a short growing season, we found differences in nektonic invertebrate resource density across wetland types and a positive relationship between duck density and energy density, contrary to previous duck food studies conducted in a similar system (Gammonley & Laubhan, 2002, although this study used a different food sampling approach). Our results suggest that duck pair numbers, but not necessarily ducklings, in given wetland types follow macroinvertebrate resource availability during times of most essential resource need and that birds are capable of locating consistent, abundant resources across the landscape (Dessborn et al., 2009). A great deal of research on breeding habitat selection among waterfowl has occurred in the prairies of North America and has evaluated wetland type (e.g., small complexes of shallow depressions versus large basins; Bloom et al., 2013; Murkin et al., 1997) as a proxy for nutrients. O'Neil et al. (2014) found that conspecific density and proximity to successful nesting habitat drove pre‐breeding habitat selection for lesser scaup more so than habitat attributes like food availability, but there is some evidence that mallards can forecast wetland conditions to a future time during which broods will need high‐quality foraging sites (Casazza et al., 2020; Pöysä et al., 2000). The sites that had higher energy density also typically had high resource stability and invertebrate taxonomic richness, suggesting that there is not necessarily a trade‐off between sites with higher energy density and those with higher temporal stability in energy (Ernest & Brown, 2001). While energy density consistently performed better in predicting waterfowl density than resource stability or taxonomic richness, further experimental research could provide deeper insight into the community compositional shifts of aquatic macroinvertebrates and the role that plays in resource stability, energy density, and subsequent waterfowl habitat use (e.g., Benoy et al., 2002).

The composition of invertebrate taxa in wetlands likely played a role in each habitat's energy density and temporal stability. In our study, wetlands that harbored the most taxonomically diverse macroinvertebrate resources typically also held the highest energy densities (Figure 4), indicating that pulses in resources may be related to the taxonomic diversity of those resources. Although an evaluation of the drivers and phenology of different taxa was beyond the scope of this study, we did examine the proportion of each sample comprised by each taxon using both number of individuals and energy density (Appendix S1). Two of the most frequently observed taxa in this study were Daphnia spp. (water fleas) and Ostracods (seed shrimp), both in terms of relative abundance and energy density. While these invertebrates can prove too small for efficient filtering by some duck species (Collias & Collias, 1963), they are commonly ingested by others and readily available in most wetland types (DuBowy, 1997; Rogers & Korschgen, 1966; Swanson, 1977). Importantly, these taxa, and Daphnia in particular, comprised the largest proportion of invertebrates in reservoirs and basin wetlands and were responsible for the sole pulse in energy density observed in basin wetlands on occasions two and three, along with Culicidae larvae (Figure 2, Appendix S1). Gammaridae (scuds) were also important contributors to resource density in basin wetlands in the first two sampling occasions. Consequently, basins and reservoirs were also associated with the most ducks by absolute number and by density, and the pulse in energy in occasions one and two coincides with the evolved migration arrival phenology of breeding ducks and nest site prospecting (Dessborn et al., 2009; Eichholz & Elmberg, 2014; Krapu & Reinecke, 1992).

Wetlands associated with flood‐irrigated agriculture exhibited both lower macroinvertebrate resource density and fewer observed waterfowl. Irrigation ditches hold flowing water that has been recently redirected from rivers, and hay meadows are typically engineered to have inflows and outflows so water is continuously moving through the system (Tate et al., 2005). These attributes may result in higher dissolved oxygen and lower water temperatures. In addition, hay meadows are typically a monoculture of Timothy grass (*Phleum pretense*), which is short in stature early in the growing season (because it is cut near the end of the previous growing season) and does not provide a diverse substrate on which macroinvertebrates might feed and develop (Fredrickson & Reed, 1988; Harrison et al., 2017). Waterfowl have been shown to avoid hay meadows when selecting a nesting site (Setash, 2023) and may be cueing in on the lack of available macroinvertebrate resources as one of their selection criteria. Hay meadows occasionally harbored resource densities comparable to those in more semi‐permanent wetland types in our study and others (e.g., Janke et al., 2019), but the rapidly changing water levels may have resulted in more ephemeral resource availability that ducks found more difficult to exploit, in addition to suboptimal nesting locations (Setash, 2023). Semi‐permanent basin wetlands and reservoirs, on the other hand, have hydrologies that encourage the growth of submerged aquatic vegetation (SAV), which provide growth and reproduction substrates for macroinvertebrates (Batzer, 2013; Bauer et al., 2020; Fredrickson & Reed, 1988; Hagy et al., 2011; Schad et al., 2020). Further research on both the physical and chemical properties of working wetlands and how they might further impact patterns of invertebrate distribution are warranted (Arzel et al., 2020; Kantrud, 1986; Longcore et al., 2006; Swanson et al., 1988; Vargas et al., 2022; Vest et al., 2023).

The amount of food in a given wetland is not always directly proportional to the observed density of waterfowl using that wetland, and many components of the habitat may preclude waterfowl from freely distributing themselves according to food availability (Brasher et al., 2007; Hagy & Kaminski, 2015; Holopainen et al., 2024; Paasivaara & Pöysä, 2008). In this system, agricultural wetlands were flooded and dried according to production needs, often resulting in dry, mowed fields during the peak of brood‐rearing (Duebbert & Frank, 1984; McVey, 2011). In contrast, reservoirs and basin wetlands consistently held water toward the end of the breeding season when broods congregated to forage. Semi‐permanent wetlands also typically had more open water, which may have allowed birds to use observable SAV as an indication that a given wetland may provide food for their ducklings and find refuge in large water bodies away from edges and dense cover (Behney et al., 2018; Fredrickson & Reed, 1988; Holopainen et al., 2024). Indeed, although energy density best predicted duckling density of all focal species but gadwall (Table 2), the relationships were negative and imprecise (Figure 7). This suggests that there are likely unmeasured drivers of wetland selection among some duck species with broods that are more important than invertebrate resources during this time (Holopainen et al., 2015). Water availability, safety, conspecific habitat use (Pöysä et al., 1998), or unmeasured cover characteristics are all likely playing a role in habitat selection by breeding birds and brooding hens, and likely vary over the course of the breeding season. Predator avoidance is of particular importance to habitat selection during the vulnerable brood‐rearing period, when hens are often simultaneously molting and both are therefore susceptible to predators (Hohman et al., 1992; Ringelman, 1990). Noninvertebrate wetland characteristics may also be important drivers of habitat selection within wetlands of a given type wherein invertebrate density is asymptotically high (e.g., basins). For example, birds likely needed more protein soon after arriving on the breeding grounds and may have found that protein in early‐thawing semi‐permanent wetlands (Murkin & Kadlec, 1986; Schepker et al., 2019; Tidwell et al., 2013). After initiating nests, however, birds may have prioritized foraging in wetlands closer to their nest sites, wetlands with lower predation risk, or relied on body reserves more so than taking frequent foraging trips. Additionally, density‐dependent processes may be masking latent selection mechanisms or limiting the plasticity of habitat selection (Nummi et al., 2015). The different foraging preferences exhibited by nesting hens over the course of laying and nest initiation are primed for further research and may elucidate mechanisms driving reproductive success.

As weather and precipitation patterns become more variable and water becomes increasingly limiting, having an understanding about which habitats provide food for wetland‐dependent species will become more important (Skagen et al., 2016; Zhao et al., 2019). The results of our study suggest that providing food‐dense wetland resources promotes use by breeding waterfowl, and that these resources may be especially important during the very early breeding season, when pre‐nesting birds are preparing to nest. The patterns of observed resource density and duck density within basin wetlands and reservoirs suggest that having the infrastructure to move water between habitat types, and to prioritize these types of wetlands during dry years, may prove essential to maintaining stable breeding populations of waterfowl across the semiarid West (Downard et al., 2014; Downard & Endter‐Wada, 2013; Sueltenfuss et al., 2013). In addition, those wetland types may be particularly important habitats on working lands where most wetland habitat is associated with agricultural production. Still, periodic drying of wetlands within this system maintains long‐term productivity and emphasizes the importance of diverse wetland types and hydrologies (Fredrickson, 1991). Breeding waterfowl take advantage of ephemeral resources across their annual cycle and they appear to be adept at locating those ephemeral resources. The results of our study suggest there is not a consequential trade‐off to selecting wetland sites based on energy density versus temporal resource stability, but rather that good‐quality wetland sites provide both. The results of this study have the potential to inform wetland restoration practices across arid landscapes in addition to the mechanisms driving habitat selection decisions of breeding waterfowl.

## AUTHOR CONTRIBUTIONS

**Casey M. Setash:** Conceptualization (equal); data curation (lead); formal analysis (lead); funding acquisition (supporting); investigation (lead); methodology (lead); project administration (lead); visualization (lead); writing – original draft (lead); writing – review and editing (lead). **Adam C. Behney:** Conceptualization (supporting); formal analysis (supporting); funding acquisition (supporting); methodology (supporting); resources (supporting). **James H. Gammonley:** Conceptualization (supporting); funding acquisition (supporting); resources (lead); supervision (supporting); writing – review and editing (supporting). **David N. Koons:** Conceptualization (equal); formal analysis (supporting); funding acquisition (lead); investigation (equal); methodology (supporting); resources (equal); supervision (lead); writing – original draft (supporting); writing – review and editing (supporting).

## CONFLICT OF INTEREST STATEMENT

The authors have no conflicts of interest to declare.

## Supporting information


Appendix S1.

## Data Availability

Many of the data used in this study are available at the end of this document as appendices, but more detailed data are available at the following location: https://doi.org/10.5061/dryad.2280gb61c. Reviewer URL: https://datadryad.org/stash/share/6Mp1VS9k2U7dpVaPTn1HnUuEO1nY4U43dVH1bGBovWc.
