# Supplementary material for: Riding the wetland wave: Can ducks locate macroinvertebrate resources across the breeding season?
Source: Ecol Evol. 2024 Jun 25;14(6):e11568. doi: 10.1002/ece3.11568 (PMC11199343; doi:10.1002/ece3.11568)
Supplement: Supplementary file 1 — Appendix S1. [file ECE3-14-e11568-s001.zip › ece311568-sup-0001-Supinfo01.docx]

**Table S1:** All taxonomic groups identified in the invertebrate samples from North Park, Colorado during May-July, 2020 and 2021. If some taxonomic groups are nested within one another, it indicates that the individuals marked in the higher taxonomic group could not be identified further. Total number of individuals observed refers to the total number counted or the total number estimated via subsampling methods described in the main text.

| **Lowest Taxonomic Classification Identified** | **Habitat(s) Observed** | **Year(s) Observed** | **Total No. Individuals Observed** |
| --- | --- | --- | --- |
| Acalyptratae | Basin, Reservoir, Riparian | 2021 | 13 |
| Aeshnidae | Basin, Ditch, Reservoir, Riparian | 2021 | 13 |
| Ameletidae | Basin, Ditch, Riparian | 2020, 2021 | 5 |
| Ancylidae | Riparian | 2020 | 1 |
| Aphididae | Reservoir | 2021 | 2 |
| Aranea | Basin, Ditch, Hay, Reservoir, Riparian | 2020, 2021 | 109 |
| Asellidae | Basin, Ditch, Reservoir, Riparian | 2020, 2021 | 27 |
| Athericidae | Hay | 2021 | 1 |
| Baetidae | Basin, Ditch, Hay, Reservoir, Riparian | 2020, 2021 | 1079 |
| Bivalvia | Ditch, Riparian | 2021 | 20 |
| Brachycentridae | Basin, Ditch, Reservoir, Riparian | 2020, 2021 | 15 |
| Caenidae | Basin, Reservoir | 2020, 2021 | 12 |
| Calliphoridae | Basin | 2021 | 1 |
| Calyptratae | Reservoir | 2021 | 2 |
| Cantharidae | Basin | 2021 | 2 |
| Carabidae | Basin, Ditch, Reservoir | 2020 | 4 |
| Cerambycidae | Basin, Riparian | 2021 | 3 |
| Ceratopogonidae | Basin, Ditch, Hay, Reservoir, Riparian | 2020, 2021 | 219 |
| Cercopidae | Basin, Riparian | 2021 | 2 |
| Chirocephalidae | Basin, Ditch, Hay, Reservoir, Riparian | 2020, 2021 | 516 |
| Chironomidae | Basin, Ditch, Hay, Reservoir, Riparian | 2020, 2021 | 2502 |
| Chloroperlidae | Basin, Hay, Riparian | 2020, 2021 | 7 |
| Chrysomelidae | Reservoir | 2021 | 1 |
| Cicadellidae | Basin, Ditch, Hay, Reservoir, Riparian | 2020, 2021 | 73 |
| Coenagrionidae | Basin, Ditch, Hay, Reservoir, Riparian | 2020, 2021 | 291 |
| Collembola | Basin, Ditch, Hay, Reservoir, Riparian | 2021 | 782 |
| Corixidae | Basin, Ditch, Hay, Reservoir, Riparian | 2020, 2021 | 7445 |
| Culicidae | Basin, Ditch, Hay, Reservoir, Riparian | 2020, 2021 | 11543 |
| Curculionidae | Basin, Ditch, Hay, Reservoir, Riparian | 2020, 2021 | 130 |
| Cynipoidea | Basin, Ditch, Reservoir, Riparian | 2021 | 14 |
| Daphniidae | Basin, Ditch, Hay, Reservoir, Riparian | 2020, 2021 | 204589 |
| Delphacidae | Basin | 2021 | 1 |
| Dermestidae | Ditch | 2021 | 1 |
| Diaptomidae | Basin, Ditch, Hay, Reservoir, Riparian | 2020, 2021 | 8709 |
| Dixidae | Basin, Reservoir | 2021 | 3 |
| Dolichopodidae | Reservoir, Riparian | 2021 | 5 |
| Dysticidae | Basin, Ditch, Hay, Reservoir, Riparian | 2020, 2021 | 7093 |
| Elmidae | Basin, Ditch, Hay, Reservoir, Riparian | 2020, 2021 | 271 |
| Empididae | Reservoir | 2021 | 2 |
| Ephemeroptera | Basin, Ditch, Hay, Reservoir, Riparian | 2020, 2021 | 74 |
| Ephemerellidae | Ditch, Riparian | 2021 | 6 |
| Fanniidae | Basin | 2021 | 11 |
| Forficulidae | Ditch | 2021 | 1 |
| Formicidae | Basin, Ditch, Hay, Reservoir, Riparian | 2020, 2021 | 64 |
| Gammaridae | Basin, Ditch, Hay, Reservoir, Riparian | 2020, 2021 | 17337 |
| Geometridae | Riparian | 2021 | 1 |
| Gerridae | Basin, Ditch, Hay, Reservoir, Riparian | 2020, 2021 | 26 |
| Gyrinidae | Basin, Ditch, Hay, Reservoir, Riparian | 2020, 2021 | 436 |
| Haliplidae | Basin, Ditch, Hay, Reservoir, Riparian | 2020, 2021 | 1670 |
| Hebridae | Basin, Riparian | 2020, 2021 | 2 |
| Helophoridae | Basin, Ditch, Hay, Reservoir, Riparian | 2020, 2021 | 365 |
| Hemiptera – unknown family | Basin, Ditch, Reservoir, Riparian | 2021 | 28 |
| Heptageniidae | Basin, Hay, Riparian | 2020, 2021 | 5 |
| Hirundinea | Basin, Ditch, Hay, Reservoir, Riparian | 2020, 2021 | 682 |
| Hydrachnidia | Basin, Ditch, Hay, Reservoir, Riparian | 2020, 2021 | 3835 |
| Hydrophilidae | Basin, Ditch, Hay, Reservoir, Riparian | 2020, 2021 | 981 |
| Hydropsychidae | Basin, Reservoir | 2021 | 4 |
| Hydrozoa | Ditch, Reservoir, Riparian | 2021 | 9 |
| Ichneumonidae | Basin, Ditch, Hay, Reservoir, Riparian | 2020 | 27 |
| Isopoda | Reservoir | 2021 | 1 |
| Isotomidae | Basin, Ditch, Hay, Reservoir, Riparian | 2020 | 939 |
| Ixodidae | Basin, Ditch, Hay, Reservoir, Riparian | 2020, 2021 | 21 |
| Lepidostomatidae | Basin, Reservoir, Riparian | 2020, 2021 | 11 |
| Leptoceridae | Basin, Hay, Reservoir, Riparian | 2020, 2021 | 18 |
| Leptohyphidae | Basin, Riparian | 2020 | 3 |
| Leptophlebiidae | Basin, Ditch, Reservoir, Riparian | 2020, 2021 | 55 |
| Lestidae | Basin, Ditch, Reservoir, Riparian | 2020, 2021 | 67 |
| Leuctridae | Riparian | 2021 | 1 |
| Libellula | Basin, Ditch, Hay, Reservoir, Riparian | 2020, 2021 | 29 |
| Limnephilidae | Basin, Ditch, Hay, Reservoir, Riparian | 2020, 2021 | 75 |
| Lymnaeidae | Basin, Ditch, Hay, Reservoir, Riparian | 2020, 2021 | 437 |
| Meloidae | Ditch, Riparian | 2020, 2021 | 3 |
| Miridae | Basin, Ditch, Hay, Reservoir, Riparian | 2020, 2021 | 14 |
| Muscidae | Basin, Ditch, Hay, Riparian | 2021 | 9 |
| Nematoda | Basin, Ditch, Hay, Reservoir, Riparian | 2021 | 1088 |
| Nemouridae | Reservoir, Riparian | 2021 | 4 |
| Noctuidae | Basin | 2020 | 2 |
| Notonectidae | Basin, Ditch, Hay, Reservoir, Riparian | 2020, 2021 | 234 |
| Odontoceridae | Ditch, Riparian | 2020, 2021 | 3 |
| Oligochaeta | Basin, Ditch, Hay, Reservoir, Riparian | 2021 | 286 |
| Ostracoda | Basin, Ditch, Hay, Reservoir, Riparian | 2020, 2021 | 178443 |
| Palaemonidae | Basin, Reservoir | 2020, 2021 | 8 |
| Parasitoid Wasp | Basin, Ditch, Hay, Reservoir, Riparian | 2021 | 20 |
| Pentatomidae | Ditch, Riparian | 2020, 2021 | 2 |
| Perlidae | Ditch | 2021 | 1 |
| Perlodidae | Ditch, Riparian | 2020, 2021 | 4 |
| Phoridae | Reservoir, Riparian | 2021 | 4 |
| Phryganeidae | Basin, Ditch, Reservoir, Riparian | 2020, 2021 | 23 |
| Physidae | Basin, Ditch, Hay, Reservoir, Riparian | 2020, 2021 | 234 |
| Planorbidae | Basin, Ditch, Hay, Reservoir, Riparian | 2020, 2021 | 408 |
| Plecoptera | Basin, Ditch, Hay, Reservoir, Riparian | 2020, 2021 | 42 |
| Polycentropodidae | Basin, Hay, Reservoir, Riparian | 2020, 2021 | 19 |
| Psychodidae | Basin, Ditch, Riparian | 2021 | 6 |
| Ptchopteridae | Basin, Hay, Reservoir, Riparian | 2021 | 12 |
| Scathophagidae | Basin, Ditch, Riparian | 2021 | 4 |
| Sciaridae | Basin, Reservoir, Riparian | 2021 | 12 |
| Silphidae | Basin | 2021 | 19 |
| Simuliidae | Basin, Ditch, Hay, Reservoir, Riparian | 2020, 2021 | 4615 |
| Siphlonuridae | Basin, Ditch, Riparian | 2020, 2021 | 81 |
| Sphaeriidae | Basin, Ditch, Hay, Riparian | 2020, 2021 | 55 |
| Staphylinidae | Basin, Ditch, Hay, Reservoir, Riparian | 2020, 2021 | 129 |
| Stratomyidae | Basin, Hay, Reservoir | 2021 | 4 |
| Tabanidae | Hay, Reservvoir | 2021 | 2 |
| Taeniopterygidae | Ditch, Riparian | 2021 | 4 |
| Tardigrada | Basin, Riparian | 2021 | 3 |
| Tenebrionidae | Hay, Reservoir, Riparian | 2021 | 4 |
| Thysanoptera | Basin, Ditch, Hay, Reservoir, Riparian | 2020, 2021 | 40 |
| Tipulidae | Hay, Reservoir, Riparian | 2021 | 11 |
| Trematoda | Basin, Ditch, Hay, Reservoir, Riparian | 2021 | 2430 |
| Trichoptera | Basin, Ditch, Hay, Reservoir, Riparian | 2020, 2021 | 96 |
| Triopsidae | Basin, Ditch, Hay | 2020 | 31 |
| Uenoidae | Reservoir, Riparian | 2021 | 5 |
| Unionida | Basin | 2021 | 1 |
| Veliidae | Basin, Ditch, Hay, Reservoir, Riparian | 2020, 2021 | 7 |

**Table S2:** Summary of pair counts conducted in North Park, CO, USA in 2020-2021 according to year, habitat type, and species. The proportion of the total count refers to the proportion of the total number of observed ducks in a given habitat and year comprised of the species in that row. CITE = cinnamon teal, GADW = gadwall, LESC = lesser scaup, and MALL = mallard.

| **Year** | **Habitat** | **Species** | **Proportion of Total Count** |
| --- | --- | --- | --- |
| 2020 | Basin | CITE | 0.2 |
| 2020 | Basin | GADW | 0.34 |
| 2020 | Basin | LESC | 0.18 |
| 2020 | Basin | MALL | 0.28 |
| 2020 | Ditch | CITE | 0.27 |
| 2020 | Ditch | GADW | 0.13 |
| 2020 | Ditch | LESC | 0 |
| 2020 | Ditch | MALL | 0.6 |
| 2020 | Hay | CITE | 0.26 |
| 2020 | Hay | GADW | 0.14 |
| 2020 | Hay | LESC | 0 |
| 2020 | Hay | MALL | 0.59 |
| 2020 | Reservoir | CITE | 0.04 |
| 2020 | Reservoir | GADW | 0.49 |
| 2020 | Reservoir | LESC | 0.21 |
| 2020 | Reservoir | MALL | 0.25 |
| 2020 | Riparian | CITE | 0.11 |
| 2020 | Riparian | GADW | 0.11 |
| 2020 | Riparian | LESC | 0 |
| 2020 | Riparian | MALL | 0.78 |
| 2021 | Basin | CITE | 0.06 |
| 2021 | Basin | GADW | 0.49 |
| 2021 | Basin | LESC | 0.11 |
| 2021 | Basin | MALL | 0.34 |
| 2021 | Ditch | CITE | 0 |
| 2021 | Ditch | GADW | 0 |
| 2021 | Ditch | LESC | 0 |
| 2021 | Ditch | MALL | 1 |
| 2021 | Hay | CITE | 0 |
| 2021 | Hay | GADW | 0 |
| 2021 | Hay | LESC | 0 |
| 2021 | Hay | MALL | 1 |
| 2021 | Reservoir | CITE | 0.11 |
| 2021 | Reservoir | GADW | 0.45 |
| 2021 | Reservoir | LESC | 0.14 |
| 2021 | Reservoir | MALL | 0.3 |
| 2021 | Riparian | CITE | 0.14 |
| 2021 | Riparian | GADW | 0.09 |
| 2021 | Riparian | LESC | 0 |
| 2021 | Riparian | MALL | 0.76 |

**Table S3:** Summary of brood counts conducted in North Park, CO, USA in 2020-2021 according to year, habitat type, and species. The proportion of the total count refers to the proportion of the total number of observed ducklings in a given habitat and year comprised of the species in that row. CITE = cinnamon teal, GADW = gadwall, LESC = lesser scaup, and MALL = mallard.

| Year | Habitat | Species | Proportion of Total Count |
| --- | --- | --- | --- |
| 2020 | Basin | CITE | 0.17 |
| 2020 | Basin | GADW | 0.71 |
| 2020 | Basin | LESC | 0.22 |
| 2020 | Basin | MALL | 0.02 |
| 2020 | Ditch | CITE | 0 |
| 2020 | Ditch | GADW | 0 |
| 2020 | Ditch | LESC | 0 |
| 2020 | Ditch | MALL | 0 |
| 2020 | Hay | CITE | 0 |
| 2020 | Hay | GADW | 0 |
| 2020 | Hay | LESC | 0 |
| 2020 | Hay | MALL | 0 |
| 2020 | Reservoir | CITE | 0.14 |
| 2020 | Reservoir | GADW | 0.61 |
| 2020 | Reservoir | LESC | 0.09 |
| 2020 | Reservoir | MALL | 0.22 |
| 2020 | Riparian | CITE | 0.33 |
| 2020 | Riparian | CITE | 0.33 |
| 2020 | Riparian | GADW | 0.67 |
| 2020 | Riparian | LESC | 0 |
| 2020 | Riparian | MALL | 0 |
| 2021 | Basin | CITE | 0.16 |
| 2021 | Basin | CITE | 0.16 |
| 2021 | Basin | GADW | 0.17 |
| 2021 | Basin | LESC | 0.54 |
| 2021 | Basin | MALL | 0.2 |
| 2021 | Ditch | CITE | 0 |
| 2021 | Ditch | CITE | 0 |
| 2021 | Ditch | GADW | 0 |
| 2021 | Ditch | LESC | 0 |
| 2021 | Ditch | MALL | 0 |
| 2021 | Reservoir | CITE | 0.29 |
| 2021 | Reservoir | CITE | 0.29 |
| 2021 | Reservoir | GADW | 0.4 |
| 2021 | Reservoir | LESC | 0.15 |
| 2021 | Reservoir | MALL | 0.27 |
| 2021 | Riparian | CITE | 0.5 |
| 2021 | Riparian | GADW | 0 |
| 2021 | Riparian | LESC | 0 |
| 2021 | Riparian | MALL | 0.5 |


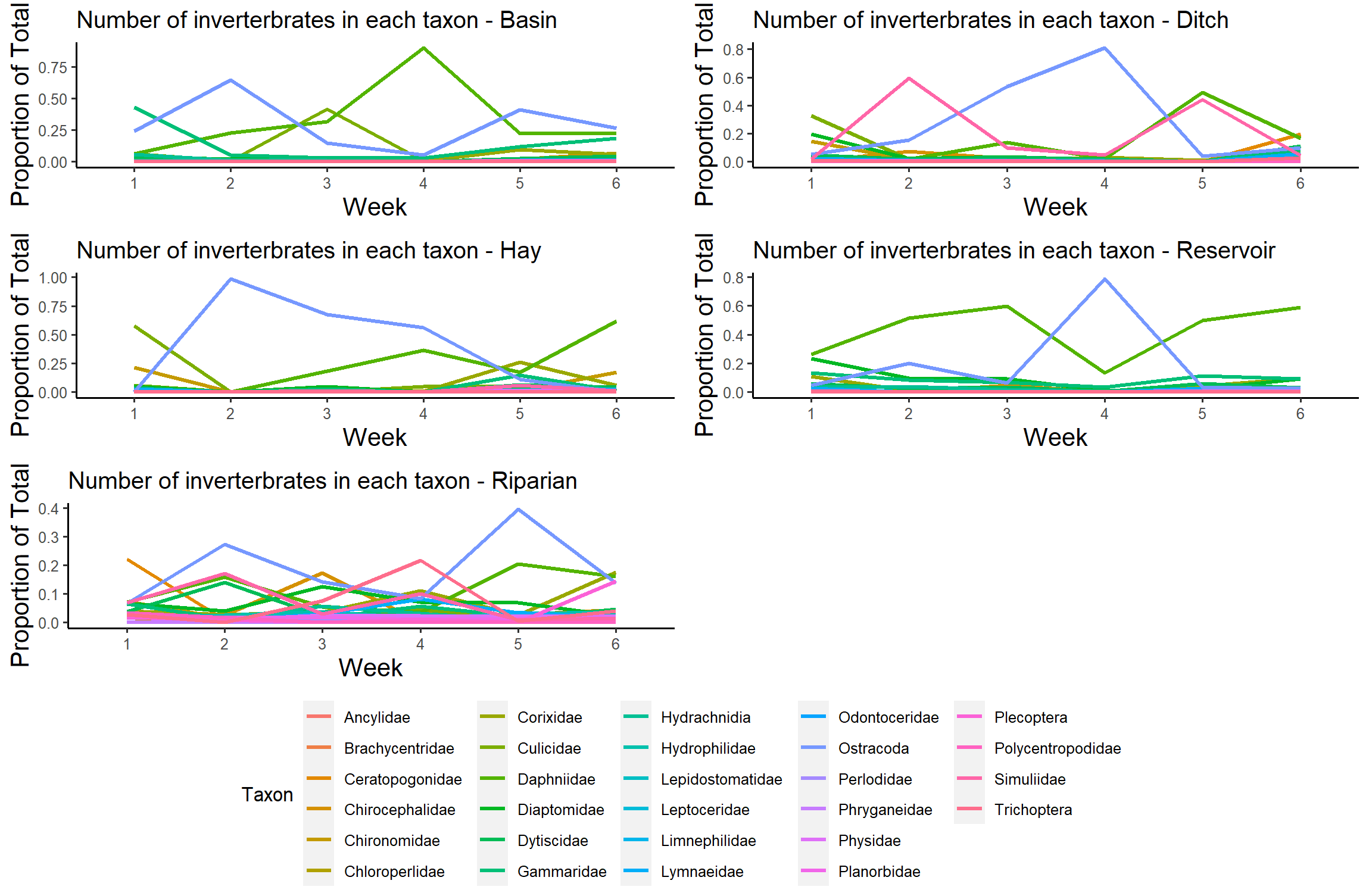


Daphnia

Daphnia

Daphnia

Ostracoda

Ostracoda

Ostracoda

Ostracoda

Ostracoda

Simuliidae

**Figure S1:** The number of invertebrates in each taxonomic family, represented as the proportion of the total number in a given sample, across sampling periods (May-July) for each wetland habitat type in North Park, CO, USA. Data are averaged across sampling years (2020-2021) and sites to depict habitat-specific trends.


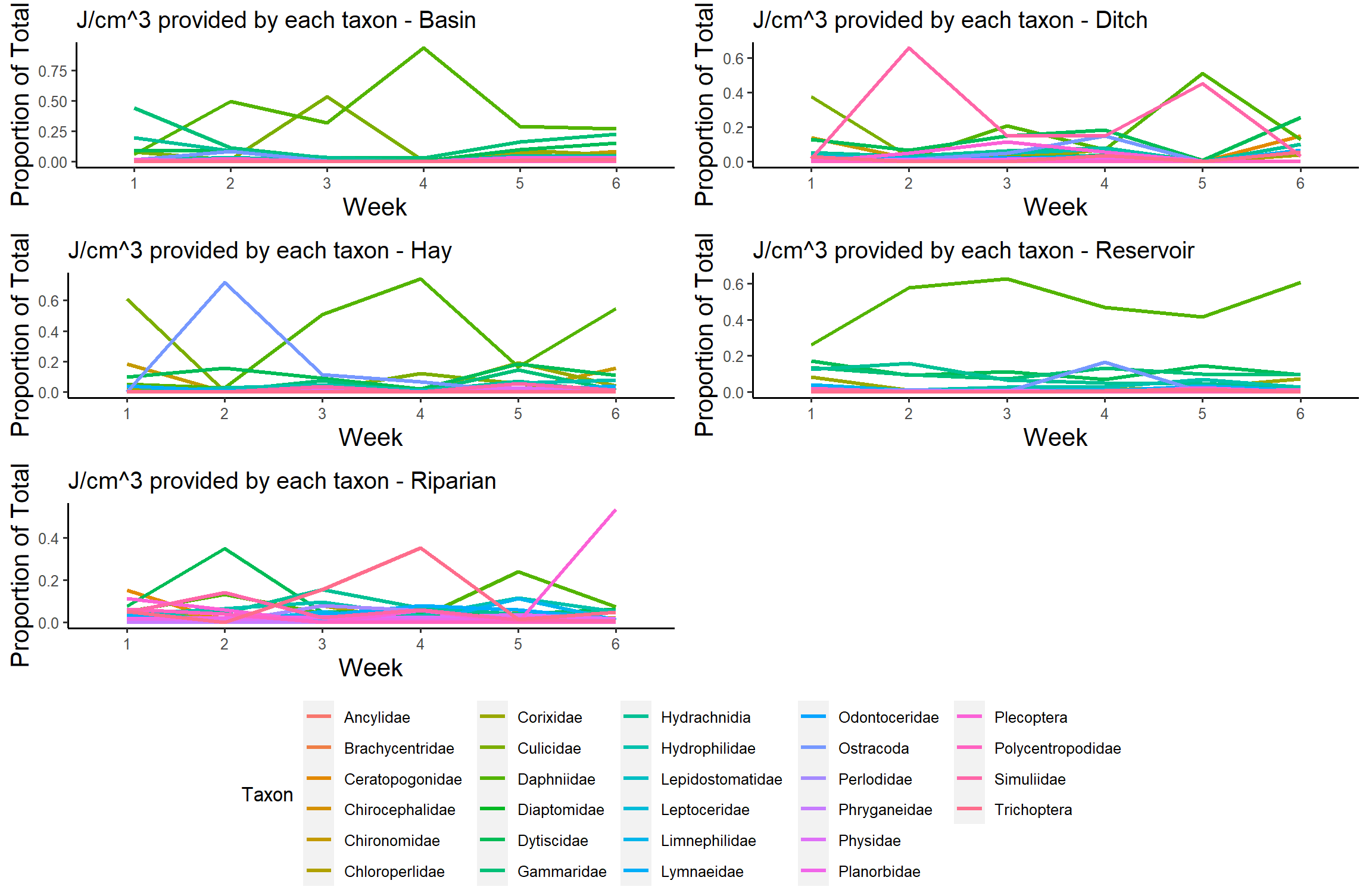


Simuliidae

Daphnia

Daphnia

Daphnia

Daphnia

Daphnia

Culicidae

Ostracoda

**Figure S2:** The energy density provided by each taxon of macroinvertebrates, represented as the proportion of the total energy density in a given sample, across sampling periods (May-July) for each wetland habitat type in North Park, CO, USA. Data are averaged across sampling years (2020-2021) and sites to look at habitat-specific trends.
